# Intraoperative frozen section performance for thyroid cancer diagnosis

**DOI:** 10.20945/2359-3997000000445

**Published:** 2022-01-01

**Authors:** Iuri Martin Goemann, Francisco Paixão, Alceu Migliavacca, José Ricardo Guimarães, Rafael Selbach Scheffel, Ana Luiza Maia

**Affiliations:** 1 Universidade Federal do Rio Grande do Sul Faculdade de Medicina Hospital de Clínicas de Porto Alegre Porto Alegre RS Brasil Unidade de Tireoide, Hospital de Clínicas de Porto Alegre, Faculdade de Medicina, Universidade Federal do Rio Grande do Sul, Porto Alegre, RS, Brasil; 2 Universidade do Vale do Rio dos Sinos Faculdade de Medicina São Leopoldo RS Brasil Faculdade de Medicina, Universidade do Vale do Rio dos Sinos, São Leopoldo, RS, Brasil; 3 Universidade Federal do Rio Grande do Sul Faculdade de Medicina Hospital de Clínicas de Porto Alegre Porto Alegre RS Brasil Divisão de Cirurgia, Hospital de Clínicas de Porto Alegre, Faculdade de Medicina, Universidade Federal do Rio Grande do Sul, Porto Alegre, RS, Brasil; 4 Universidade Federal do Rio Grande do Sul Instituto de Ciências Básicas da Saúde Departamento de Farmacologia Porto Alegre RS Brasil Departamento de Farmacologia, Instituto de Ciências Básicas da Saúde, Universidade Federal do Rio Grande do Sul, Porto Alegre, RS, Brasil

**Keywords:** Thyroid cancer, intraoperative frozen section, Bethesda classification, thyroid nodules

## Abstract

**Objective:**

A primary medical relevance of thyroid nodules consists of excluding thyroid cancer, present in approximately 5% of all thyroid nodules. Fine-needle aspiration biopsy (FNAB) has a paramount role in distinguishing benign from malignant thyroid nodules due to its availability and diagnostic performance. Nevertheless, intraoperative frozen section (iFS) is still advocated as a valuable tool for surgery planning, especially for indeterminate nodules.

**Subjects and methods:**

To compare the FNAB and iFS performances in thyroid cancer diagnosis among nodules in Bethesda Categories (BC) I to VI. The performance of FNAB and iFS tests were calculated using final histopathology results as the gold standard.

**Results:**

In total, 316 patients were included in the analysis. Both FNAB and iFS data were available for 272 patients (86.1%). The overall malignancy rate was 30.4%% (n = 96). The FNAB sensitivity, specificity, and accuracy for benign (BC II) and malignant (BC V and VI) were 89.5%, 97.1%, and 94.1%, respectively. For all nodules evaluated, the iFS sensitivity, specificity, and accuracy were 80.9%, 100%, and 94.9%, respectively. For indeterminate nodules and follicular lesions (BC III and IV), the iFS sensitivity, specificity, and accuracy were 25%, 100%, and 88.7%, respectively. For BC I nodules, iFS had 95.2% of accuracy.

**Conclusion:**

Our results do not support routine iFS for indeterminate nodules or follicular neoplasms (BC III and IV) due to its low sensitivity. In these categories, iFS is not sufficiently accurate to guide the intraoperative management of thyroidectomies. iFS for BC I nodules could be an option and should be specifically investigated

## INTRODUCTION

Thyroid nodules are common and can be detected by ultrasound (US) in 50%-60% of adults (1). Most of these lesions are benign, and only 9% to 13% of those nodules selected for fine-needle aspiration biopsy (FNAB) are diagnosed as thyroid cancer (2,3). Trend analysis reveals an increase in thyroid cancer diagnosis in the last decades, resulting from overdiagnosis and possibly also from environmental factors (4). It is noteworthy that malignant thyroid disease has been subject to a more conservative surgical approach, mainly when tumor stratification determines low-risk recurrence (5).

Due to its diagnostic performance and wide availability, the cytological analysis of the material obtained by FNAB has a paramount role in distinguishing benign from malignant thyroid nodules (6). However, FNAB has an intrinsic limitation to establish the diagnosis of follicular or Hürthle malignant cell lesions, as the demonstration of capsular or vascular invasion is required to distinguish benign from malignant non-papillary thyroid tumors (7,8). In this context, intraoperative frozen section (iFS) has been historically advocated as an essential tool in defining the extent of thyroid surgery (total vs. partial thyroidectomy).

After the introduction of the Bethesda System for Reporting Thyroid Cytopathology (BSRTC), followed by the consequent simplification of the cytopathologic diagnosis, surgical planning is mainly based on the preoperative diagnosis. However, many surgical teams still consider iFS as a useful tool to optimize the decision regarding the extent of surgery, especially for indeterminate nodules (Bethesda Categories III or IV), which represent approximately 20% of all thyroid FNAB and associated with a malignancy risk of 5%-30% (8-10). Cost-benefit analysis often associates iFS procedures with higher costs due to time, technical and human resources needed to interpret the test during surgeries accompanied by a limited performance in guiding intraoperative surgical decisions (11-13). Studies have evaluated the usefulness of iFS in intraoperative management, demonstrating the limited ancillary role of this diagnostic procedure, especially in indeterminate nodules, for which it would be considered most useful (14,15). However, retrospective analyses are usually based on the iFS test performed in selected nodules, which renders biased test performance results.

Here, we aimed to evaluate the FNA and iFS performances in thyroid nodules among all Bethesda categories, comparing both tests in an unbiased cohort where virtually all nodules were submitted to iFS analysis.

## SUBJECTS AND METHODS

### Patients and study design

All patients who underwent thyroid surgery due to nodular thyroid disease between January 2015 and December 2018 in the *Hospital de Clínicas de Porto Alegre* (HCPA) were candidates for inclusion in the study. Inclusion criteria were the availability of both iFS and final histopathological data on the Hospital registry. HCPA is a tertiary care, university-based teaching hospital in Southern Brazil. This study had the Institutional Ethics Committee approval (CAAE 75229317.0.0000.5327).

### Ultrasound (US)-guided fine-needle aspiration biopsy (FNAB)

Patients underwent US-guided FNAB in real-time. US was performed using a high-resolution ALOKA ultrasound device with a 7.5 MHz linear transducer (Tokyo, Japan) by three radiologists with broad thyroid imaging experience. The patients remained in a supine position, with slight cervical extension for better cervical region exposure. FNABs were performed with a disposable needle attached to a 10 mL disposable syringe. After the correct needle positioning in the nodule, continuous negative pressure and multidirectional movements were performed. An experienced staff pathologist performed a rapid on-site evaluation of fine-needle aspiration of all specimens to evaluate adequacy. For a thyroid FNAB specimen to be considered satisfactory, at least six groups of follicular cells were required, each group composed of at least ten cells (16,17). Immediate on-site re-aspiration was performed in cases considered inadequate for diagnosis. Six cytological slides were prepared for each patient, four of them air-dried and immediately stained by the May Grünwald Giemsa technique. The other two slides were immediately fixed in ethanol 96° and subsequently stained by the Papanicolaou technique. The residual hemorrhagic aspirate in the syringe and needle was rinsed in saline and processed for cell block processing (18). Cytological results classified nodules according to the criteria of the BSRTC into six diagnostic categories: I) Non-diagnosis or Unsatisfactory, II) Benign, III) Atypia of undetermined significance; IV) Follicular neoplasm or suspicious for follicular neoplasm; V) Suspicious for malignancy and VI) Malignant. Since our institution is a referral center for thyroid cancer treatment, some patients were submitted to US-guided FNAB in other centers and referred to us after the cancer diagnosis.

### Intraoperative frozen section

Intraoperative frozen section consists of a gross examination sampling of surgical specimens, followed by a microscopic examination of 4 to 5 micron-thick frozen sections, cut on a cryostat and transferred to a glass slide at room temperature and immediately fixed in either 80% ethanol or formalin with alcohol. The tissue was then progressively dehydrated before staining, followed by staining with hematoxylin & eosin. Besides, scraping and the smearing of the lesion surface were taken to an on-site cytology examination. Final diagnoses were reported to the surgeon in the operating room. For patients with more than one nodule, the analysis was conducted based on the most suspicious nodule.

### Statistical analysis

The clinical and laboratory data were reported as the average ± standard deviation (SD) values or median and percentiles 25 and 75 (P25-75) for continuous variables or absolute numbers and percentages for categorical variables. Comparisons of malignancy rates were performed by using McNemar's test (15).

The sensitivity and specificity of FNAB and iFS were calculated using final histopathology results as the gold standard. We calculated the Youden's J statistic test to evaluate the iFS performance as a dichotomous diagnostic test. The Youden index is a test performance measure, calculated based on test sensitivity and specificity (Youden index = sensitivity + specificity - 1). Its value ranges from 0 through 1: zero means the diagnostic test gives the same proportion of positive results for groups with and without the disease (useless test), and 1 is when the test is considered perfect, that is, there are no false-positive or false-negative results (19).

As many uncertain or inconclusive iFS reports were expected (deferred diagnosis), performance calculations were based on practical clinical reasoning as previously described (per intention diagnosis) (13). Since total thyroidectomy is usually performed based on a definitive carcinoma diagnosis in the frozen section, other frozen section diagnoses, such as follicular lesion and ‘deferred lesion,’ were considered as “negative test” as they did not contribute to decide the extent of thyroidectomy. Therefore, patients who had uncertain or inconclusive results were classified together with those presenting a negative result for malignancy on the iFS report (13,20).

The analyses were performed using the Statistical Package for Social Science Professional software version 20.0 (IBM Corp., Armonk, NY, USA). All tests were two-tailed, and a P < 0.05 was considered statistically significant.

## RESULTS

### Clinical characteristics

**Figure 1:**
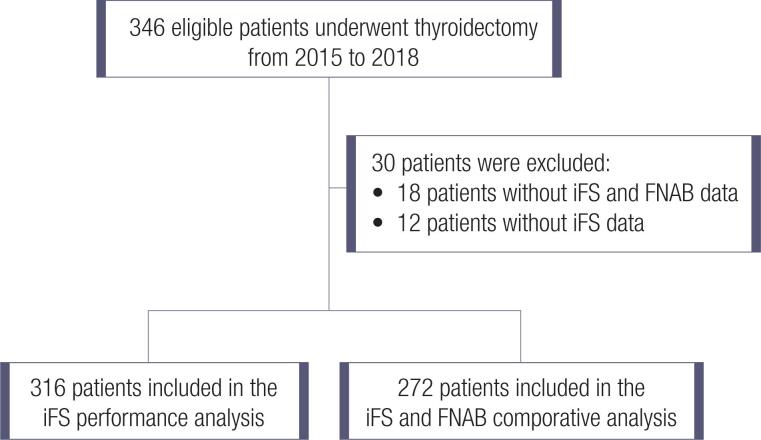
Study flow chart.

From 2015 to 2018, a total of 346 thyroidectomies due to nodular disease were performed in the HCPA. Out of these, 316 (91.3%) had iFS and pathology data and were included in the study (Figure 1). Among the study population, 275 (87%) were women, and the average age was 55.5 ± 14.4 years. The overall malignancy rate among the nodules included in the study was 26.6% (n = 84). The clinical features of the study population are summarized in Table 1. Fifteen cases of incidental carcinomas (not the index nodule) were identified in the surgical specimens. For the iFS calculation and FNAB performance, only the final histopathology of the index nodule was considered.

**Table 1:** Characteristics of the 316 patients included in the study

Age (years)		55.5 ± 14.4
Female sex – n (%)		275 (87)
FNAB result available – n (%)		272 (86)
	Bethesda I	21 (7.7)
	Bethesda II	108 (39.7)
	Bethesda III	39 (14.3)
	Bethesda IV	41 (15)
	Bethesda V	38 (14)
	Bethesda VI	25 (9.3)
Malignant nodules – n (%)		84 (26.6)
	Papillary carcinoma	68 (80.9)
	Follicular carcinoma	9 (10.8)
	Medullary carcinoma	5 (5.9)
	Poorly differentiated carcinoma	1 (1.2)
	Anaplastic carcinoma	1 (1.2)

FNAB: fine-needle aspiration biopsy.

### Preoperative thyroid nodule evaluation by FNAB

A total of 272 patients (86.1%) had preoperative FNAB data. The classification of nodules, according to the BSRTC, is reported in Table 1. Most nodules were classified as Bethesda Category (BC) II (39.7%), while nondiagnostic or unsatisfactory cytology rate (BC I) was 7.7%.

The malignancy rates in the distinct BC of nodules were: BC I (9.5%); BC II (6.5%); BC III (10.2%); BC IV (19.5%); BC V (94.7%); BC VI (96%). In BC II nodules, the BC was concordant with final histopathology results in 93.5% of nodules (n = 101); seven malignant tumors were missed. In BC V and VI nodules, the BC agreed with definitive diagnosis in 95.2% of tumors. The BSRTC performance in classifying benign (Bethesda II) and malignant (Bethesda V and VI) resulted in values for sensitivity and specificity of 89.5% and 97.1%, respectively, with an accuracy of 94.1% (Table 2, Supplementary Table 1).

**Table 2:** iFS performance in classifying thyroid nodules as benign or malignant, according to the BSRTC

Test	Sensitivity	Specificity	PPV	NPV	Accuracy
FNAB (n = 171)*	89.5%	97.1%	95.2%	93.5%	94.1%
iFS for all nodules (n = 316)	80.9%	100%	100%	93.5%	94.9%
iFS for BC I nodules (n = 21)	50%	100%	100%	95%	95.2%
iFS for BC II, V and VI nodules (n = 171)	92.5%	100%	100%	95.4%	97%
iFS for BC III and IV nodules (n = 80)	25%	100%	100%	88.3%	88.7%

PPV: positive predictive value; NPV: negative predictive value; FNAB: fine-needle aspiration biopsy; iFS: intraoperative frozen section.

* When considering BC II as a negative test and BC V and VI as a positive test, see Materials and Methods.

### Intraoperative frozen performance

Sixty-eight patients (21.5%) presented malign results on iFS and 248 (78.5%) had benign results. The final histopathology report confirmed 84 malignant and 232 benign nodules. The calculated iFS sensitivity and specificity were 80.9% and 100%, respectively, presenting an accuracy of 94.9% (Table 2, Supplementary Table 2).

The iFS accuracy in BC II, V, and VI nodules was 97%, with 92.5% and 100% sensitivity and specificity, respectively. In BC I nodules, iFS accuracy was 95.2% (Table 2, Supplementary Tables 3 and 4). For indeterminate nodules (BC III and IV), sensitivity, specificity, and accuracy were 25%, 100%, and 88.7%, respectively (Table 2, Supplementary Table 5).

### Comparison between Bethesda System classification and intraoperative frozen section in thyroid cancer diagnosis

Both FNAB and iFS data were available for 272 patients (86.1%) (Figure 1). Out of those patients who had a preoperative benign cytological FNAB (BC II, n = 108), 101 (93.5%) had nodules classified as benign and four as malignant by iFS, totalling 105 concordant results with final histology.

**Figure 2:**
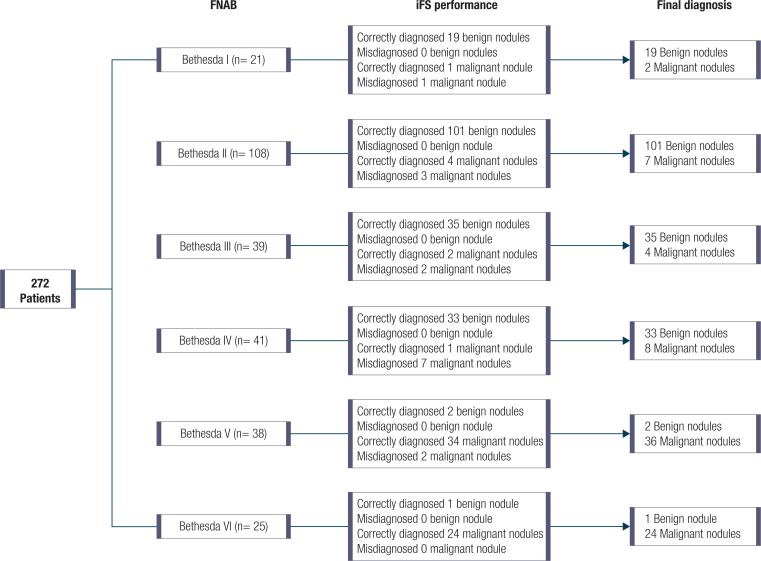
Flowchart of iFS performance for each Bethesda Category and the final histopathological diagnosis.

On the other hand, out of those who had a preoperative malignant cytological FNAB result (BC V or VI, n = 63), iFS had a concordant result with the final diagnosis in 61 patients (96.8%). Final histopathology results confirmed malignancy in 60 of these cases (95.2%) (Figure 2).

In the group of patients with a nondiagnostic or unsatisfactory FNAB (BC I, n = 21), iFS classified the lesion as benign in 19 cases (90.5%). It correctly classified one nodule as malignant, but classified as benign one malignant nodule.

Regarding patients with indeterminate FNAB results (BC III and IV, n = 80), iFS classified the lesion as benign in 77 (96.2%) and as malignant in 3 cases. However, the final histopathology results were 68 benign and 12 malignant nodules (15.6%). Thus, iFS misclassified as benign nine malignant lesions. In this group of patients, the iFS sensitivity was low (25%), although its specificity was 100%.

Therefore, by using the Youden's J statistic for iFS, we observed a high index (Yuden's index = 0.92) for nodules with benign (Bethesda II) or malign (Bethesda V or VI) preoperative. However, it is noticeable a low index in those patients who underwent diagnostic thyroidectomies (BC III and IV, Yuden's index = 0.25).

## DISCUSSION

The iFS analysis has been historically proposed as a tool for tailoring surgical extension of thyroidectomies. In the last decade, FNAB has become the most useful method to assess preoperative malignancy risk in thyroid nodules. Due to increasing thyroid cancer screening and diagnosis rates, we depend on reliable pre and intraoperative histological data to avoid overtreatment of nodular thyroid disease. In this context, we assessed the iFS performance among all BC nodules to evaluate its potential role in the surgical management of thyroid nodules. We demonstrated that the iFS accuracy is 97% in BC II, V, and VI nodules, however, when it is compared to an FNAB accuracy (94.1%), the iFS performance in BC III and IV nodules is lower, showing low sensitivity (25%) to detect malignant disease in these categories.

Studies report FNAB sensitivity ranging from 36%-89% and specificity ranging from 94-99%, presenting an accuracy from 84%-94% when considering all BC categories (10,21). However, most studies focus on cytologically indeterminate lesions (BC III and IV) (13) and include only cases in which clinical judgment guided the referral to iFS, rendering a biased selection in most analysis (22,23). We investigated routine iFS performed in our institution (94.5% of cases of nodular disease), generating a non-biased analysis concerning the iFS performance in thyroid nodules. As expected for a tertiary referral center, our cohort presents higher malignancy rates (26.6%), showing 10.2% and 19.5% of malignancy rates in BC III and IV categories. Similarly to other studies (13), the iFS accuracy was 94.9% when considering all BC nodules, a value that decreased to 88.7% in BC III and IV nodules (Table 2). Recently, Huang et al. reported an iFS accuracy rate ranging from 90-91.4% in BC I, II, V and VI nodules, but 87.9% in BC III and IV nodules. However, the cohort's malignancy rate was 84%, with most nodules in BC V and VI, which limits the interpretation of the results due to a high pre-test probability (15).

Indeed, the related iFS performed in BC III and IV nodules rendered the lowest sensitivity among the distinct BCs (25%), although showing 100% of specificity. Cotton et al. also described iFS low sensitivity, primarily for BC III and IV nodules (S = 20%) (14), which was also evident for follicular lesions in a meta-analysis (20). In BC III and IV nodules, we can observe high deferral rates in other studies (up to 68% and 84%, respectively) (11). The iFS test for BC III and IV was classified as having low utility by the Yuden's index analysis. Since deferred cases were classified as benign nodules (per intention diagnosis), we have a higher rate of misdiagnosed nodules in these categories (5.1% and 17%, respectively). In a recent meta-analysis that evaluated the iFS performance in follicular lesions, its sensitivity was also low (43%), proposing a limited utility for this test in these BC (13).

In BC V nodules, iFS misdiagnosed two malignant nodules (5.2%), whereas the Bethesda system also misclassified two nodules whose final histology was benign. Obtaining intraoperative consultation for this nodule category does not justify surgical time delay, as iFS did not change the conduct in most cases as already demonstrated by other studies (22). In Bethesda VI nodules, the iFS test was perfect. However, the high accuracy of FNAB does not support an intraoperative procedure for surgical guidance.

Although the number of patients with BC I nodules was low (n=21), iFS had a high accuracy in this group (95.2%, Table 2). Since preoperative information to determine surgery extent in this group is usually limited, the information provided by iFS could significantly help guide intraoperative management of BC nodules.

Eleven incidental papillary thyroid microcarcinomas not related to the index nodule were diagnosed in the study population. Nevertheless, according to current guidelines, thyroid lobectomy may be sufficient for the very low-risk papillary or follicular carcinomas, precluding the necessity of a complementary thyroidectomy (24). Therefore, even in this context, iFS would not significantly add information to surgical decision.

The strength of the present study is that iFS was performed in almost all nodules submitted to surgical procedure, rendering a non-biased selection analysis. As a limitation, our study could not calculate the impact of iFS on surgery time due to a lack of registered data. This calculation would be essential for cost-effectiveness analysis. A recent study evaluated the iFS cost-effectiveness for nodules with atypia of undetermined significance or follicular lesion of undetermined significance (AUS/FLUS), when its specificity was 100%, demonstrating that total thyroidectomy was avoided in one out of every 24 cases, resulting in savings of $80 per surgery in this population (25). In another analysis of BC V nodules that considered a surgical approach based on ATA 2015 guidelines, a small percentage of cases would have been converted to total thyroidectomy based on iFS. However, routine iFS would still be cost-effective if the method specificity were 100% (26), similarly for BC IV nodules, according to other studies (27,28). Nevertheless, most of the cost-effectiveness analysis does not consider operative and postoperative costs associated with unnecessary total thyroidectomies and management of complications (hypoparathyroidism, recurrent laryngeal nerve palsy), which can occur in up to 20% of the cases (29). In our study, 11.2% of patients in BC III and IV would have been submitted to unnecessary total thyroidectomy (false positive), which could significantly influence postoperative complications and their associated costs.

In conclusion our study does not support routine iFS for indeterminate nodules and follicular neoplasms (BC III and IV) due to its low sensitivity. Therefore, iFS might not be accurate enough to guide the intraoperative management of thyroidectomies in these categories, as a high rate of false positive results would be expected. Moreover, the iFS performance for BC II, V, and VI nodules is comparable to FNAB and would not significantly modify surgical management. Routine iFS in BC I nodules could improve surgical management and should be further explored.

## SUPPLEMENTARY TABLES

### Supplementary Table 1 The Bethesda system performance in nodules classified as II, V, and VI (171 nodules)

**Table:**

Bethesda result	Cancer	Benign	Test Performance
II	7	101	S: 89.5% E: 97.1%
V or VI	60	3	PPV: 95.2% NPV: 93.5%
			Accuracy: 94.1%

S: sensitivity; E: specificity; PPV: positive predictive value; NPV: negative predictive value.

### Supplementary Table 2 iFS performance in all thyroidectomies (316 nodules)

**Table:**

iFS result	Cancer	Benign	Test Performance
Benign	16	232	S: 80.9% E: 100%
Malignant	68	0	PPV: 100% NPV: 93.5%
			Accuracy: 94.9%

S: sensitivity; E: specificity; PPV: positive predictive value; NPV: negative predictive value.

### Supplementary Table 3 iFS performance in Bethesda I nodules (21 nodules)

**Table:**

iFS result	Cancer	Benign	Test Performance
Benign	1	19	S: 50% E: 100%
Malignant	1	0	PPV: 100% NPV: 95%
			Accuracy: 95.2%

S: sensitivity; E: specificity; PPV: positive predictive value; NPV: negative predictive value.

### Supplementary Table 4 iFS performance in Bethesda II, V, and VI nodules (171 nodules)

**Table:**

iFS result	Cancer	Benign	Test Performance
Benign	5	104	S: 92.5% E: 100%
Malignant	62	0	PPV: 100% NPV: 95.4%
			Accuracy: 97%

S: sensitivity; E: specificity; PPV: positive predictive value; NPV: negative predictive value.

### Supplementary Table 5 iFS performance in diagnostic thyroidectomies (Bethesda III and IV nodules) (80 nodules)

**Table:**

iFS result	Cancer	Benign	Test Performance
Benign	9	68	S: 25% E: 100%
Malignant	3	0	PPV: 100% NPV: 88.3%
			Accuracy: 88.7%

S: sensitivity; E: specificity; PPV: positive predictive value; NPV: negative predictive value.
